# Interspecific variation in cooperative burrowing behavior by *Peromyscus* mice

**DOI:** 10.1002/evl3.293

**Published:** 2022-07-22

**Authors:** Nicole L. Bedford, Jesse N. Weber, Wenfei Tong, Felix Baier, Ariana Kam, Rebecca A. Greenberg, Hopi E. Hoekstra

**Affiliations:** ^1^ Howard Hughes Medical Institute Harvard University Cambridge Massachusetts 02138 USA; ^2^ Museum of Comparative Zoology Harvard University Cambridge Massachusetts 02138 USA; ^3^ Department of Organismic and Evolutionary Biology Harvard University Cambridge Massachusetts 02138 USA; ^4^ Current address: Department of Zoology and Physiology University of Wyoming Laramie Wyoming 82071 USA; ^5^ Current address: Department of Integrative Biology University of Wisconsin–Madison Madison Wisconsin 53706 USA; ^6^ Department of Molecular and Cellular Biology Harvard University Cambridge Massachusetts 02138 USA; ^7^ Center for Brain Science Harvard University Cambridge Massachusetts 02138 USA

**Keywords:** Animal architecture, burrow, cooperation, deer mouse, social cohesion

## Abstract

Animals often adjust their behavior according to social context, but the capacity for such behavioral flexibility can vary among species. Here, we test for interspecific variation in behavioral flexibility by comparing burrowing behavior across three species of deer mice (genus *Peromyscus*) with divergent social systems, ranging from promiscuous (*Peromyscus leucopus* and *Peromyscus maniculatus*) to monogamous (*Peromyscus polionotus*). First, we compared the burrows built by individual mice to those built by pairs of mice in all three species. Although burrow length did not differ in *P. leucopus* or *P. maniculatus*, we found that *P. polionotus* pairs cooperatively constructed burrows that were nearly twice as long as those built by individuals and that opposite‐sex pairs dug longer burrows than same‐sex pairs. Second, to directly observe cooperative digging behavior in *P. polionotus*, we designed a burrowing assay in which we could video‐record active digging in narrow, transparent enclosures. Using this novel assay, we found, unexpectedly, that neither males nor females spent more time digging with an opposite‐sex partner. Rather, we demonstrate that opposite‐sex pairs are more socially cohesive and thus more efficient digging partners than same‐sex pairs. Together, our study demonstrates how social context can modulate innate behavior and offers insight into how differences in behavioral flexibility may evolve among closely related species.

Although some innate behaviors are invariantly expressed, others are labile, allowing animals to flexibly adjust their behavior according to context. Notably, this capacity for behavioral flexibility can vary among closely related species. For example, the same behavior may be fixed in one species but flexible in another (Kappeler et al. 2013; Royle et al. 2014). Such interspecific variation raises the possibility that behavioral flexibility itself can be heritable and therefore subject to natural selection. Thus, understanding the conditions under which selection may favor the fixed or flexible expression of innate behavior has been of longstanding interest in evolutionary biology (Mayr 1974).

One hypothesis is that differences in social system contribute to interspecific variation in the capacity for behavioral flexibility (Bond et al. 2007; Amici et al. 2008). Specifically, species that vary in social organization, mating system, and/or parental care strategy experience persistent differences in social environment that may promote or constrain the evolution of flexible behavior. Within species, how the social environment affects the expression of innate (i.e., unlearned) behavior is increasingly well understood. For example, social context—particularly group size—is known to modulate innate behaviors, such as odor preference in flies (Ramdya et al. 2015), mating behavior in water striders (Montiglio et al. 2017), and exploratory behavior in zebrafish (Guayasamin et al. 2017). Similarly, past social experience can alter territoriality in flies (Hoffmann 1990; Watanabe et al. 2017), social competence in cichlids (Taborsky et al. 2012), aggression in mice (Nelson et al. 2013; Stagkourakis et al. 2020), and nest building in rats (Sharpe 1975). Thus, although social context can be an important driver of behavioral flexibility within species, how the capacity for behavioral flexibility varies among species remains less clear.

Comparisons of closely related species with different social systems may be especially informative for understanding the role of social environment (if any) in the evolution of behavioral flexibility. Here, we focused on the effect of social context on burrowing behavior in three closely related species of deer mice (genus *Peromyscus*) with divergent social systems. The oldfield mouse (*Peromyscus polionotus*) is both socially and genetically monogamous (Foltz 1981), provides biparental care (Bendesky et al. 2017), and commonly nests in opposite‐sex pairs (Blair 1951). By contrast, its sister species, the deer mouse (*Peromyscus maniculatus*), and an outgroup species, the white‐footed mouse (*Peromyscus leucopus*), are highly promiscuous (Xia and Millar 1991; Ribble and Millar 1996), provide uniparental care (Xia and Millar 1988; Bendesky et al. 2017), and most often nest solitarily (Wolff and Hurlbutt 1982). Importantly, all three species construct burrows that can be used to avoid predators, cache food, or rear young.

Across *Peromyscus*, burrow architecture is both species‐specific and innate. For example, animals reared in captivity for several generations, without exposure to soil or opportunity to burrow, recapitulate their species‐specific burrow architecture when tested in the lab (Weber and Hoekstra 2009; Weber et al. 2013). Moreover, interspecific cross‐fostering shows that mice raised by another *Peromyscus* species dig burrows typical of their own species, rather than those of their foster parents (Metz et al. 2017). These data are consistent with a strong genetic basis for interspecific variation in *Peromyscus* burrowing behavior. In both the lab and the field, *P. polionotus* builds “complex” burrows consisting of a long entrance tunnel, nest chamber, and upward sloping escape tunnel, whereas *P. maniculatus* and *P. leucopus* build “simple” burrows with a short entrance tunnel and terminal nest chamber (Dawson et al. 1988; Weber and Hoekstra 2009; Weber et al. 2013). However, it is unknown whether these species‐specific architectures are fixed, or if mice flexibly adjust their burrow length according to social context.

Here, under controlled laboratory conditions, we tested mice from three *Peromyscus* species to determine if social context (i.e., digging alone or in pairs) affects burrow length. Because social system differs among these species, we may expect the ability to flexibly alter burrow length according to social context to differ as well. Specifically, we predicted that the monogamous *P. polionotus* would adjust its burrowing behavior based on social context, whereas the promiscuous *P. maniculatus* and *P. leucopus* would not. We found that the ability to alter burrow length according to social context varies among species: burrow length was unchanged between individual and pair trials in *P. maniculatus* and *P. leucopus*, whereas *P. polionotus* pairs cooperatively constructed burrows that were nearly twice as long as those dug by individuals. Moreover, by directly measuring digging behavior in the cooperatively burrowing species (*P. polionotus*), we show that digging efficiency and burrow length depend on the sex of one's digging partner. Together, these results underscore how social context can differentially affect the expression of an innate behavior, even among closely related species.

## Methods

Please see the Supporting Information.

## Results

### EFFECT OF SOCIAL CONTEXT ON BURROW LENGTH

To investigate the effect of social context on burrowing behavior, we first compared the burrows built by individual mice to those built by pairs of mice in three *Peromyscus* species. To do so, we introduced either one or two mice into large, sand‐filled enclosures. After two overnight periods, we removed the mice and made casts of the resultant burrows with polyurethane filling foam. For pair trials, we noted whether the two mice were found in the same or separate burrows at the end of the trial. We then measured each burrow cast to determine burrow length and shape. For each trial, we recorded the number of burrows, measured the length of each burrow, and categorized the shape of each burrow as either “simple” or “complex.” We further noted the length of the longest burrow produced each trial, or “maximum burrow length” (see *Methods* in the Supporting Information).

**Figure 1:**
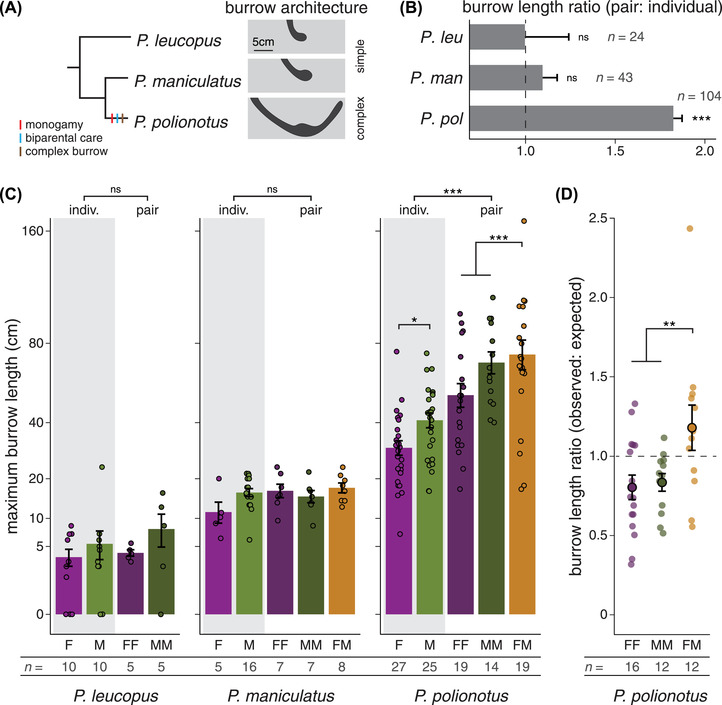
*Peromyscus polionotus* pairs cooperatively construct long burrows. (A) Phylogenetic relationships, presence/absence of derived traits, and species‐typical burrow architectures of three *Peromyscus* species. (B) Ratio of pair to individual burrow lengths in three species. A ratio >1 indicates that pairs dug longer burrows than individuals. All pair‐types (FF, MM, FM) and both sexes (F, M) are included in ratio calculations (*P. leucopus*: *n* = 9 pairs, 15 individuals; *P. maniculatus*: *n* = 22 pairs, 21 individuals; *P. polionotus*: *n* = 52 pairs, 52 individuals). (C) Maximum burrow length dug by individuals (F = purple, M = green) and pairs of mice (FF = dark purple, MM = dark green, FM = gold) in three species. Data are plotted on a square‐root scale. Data points represent the mean of 1‐7 trials per individual or unique pair. (D) Ratio of observed to expected burrow lengths in *P. polionotus* pairs, given the known output of individuals comprising the pair. *P < 0.05, **P < 0.01, ***P < 0.001. Error bars represent SEM.

In both individual and pair trials, mice from all three species produced species‐typical burrows. *Peromyscus leucopus* and *P. maniculatus* dug “simple” burrows with a short entrance tunnel and terminal nest chamber, whereas *P. polionotus* dug “complex” burrows with a long entrance tunnel, nest chamber, and escape tunnel (Fig. 1A). However, using a linear mixed‐effects model (LMM) controlling for mouse ID and sex, we found that pairs dug more burrows than individuals (individuals: 1.15 ± 0.04 burrows, pairs: 1.54 ± 0.07 burrows) in all three species (Fig. S1A; LMM, species: *F* = 2.38, *P* = 0.098; context: *F* = 31.32, *P* < 0.001; species × context: *F* = 1.38, *P* = 0.253). Nonetheless, two mice were found together in the same burrow in 58% of *P. leucopus* trials, 88% of *P. maniculatus* trials, and 100% of *P. polionotus* trials. In addition, maximum burrow length differed significantly among species and between trial‐types (Fig. S1B; LMM, species: *F* = 157.41, *P* < 0.001; context: *F* = 21.56, *P* < 0.001; species × context: *F* = 20.51, *P* < 0.001). Using planned contrasts, we found an effect of social context on maximum burrow length only in *P. polionotus* (Fig. 1B; *P. leucopus*: *t* = 0.16, *P* = 0.871; *P. maniculatus*: *t* = 0.89, *P* = 0.374; *P. polionotus*: *t* = 11.27, *P* < 0.001). *Peromyscus polionotus* pairs dug, on average, 82% longer burrows than *P. polionotus* individuals (individuals: 35.3 ± 2.1 cm, pairs: 64.3 ± 4.2 cm). By contrast, in *P. leucopus* and *P. maniculatus*, the maximum burrow length in individual and pair trials was statistically indistinguishable (*P. leucopus* individuals: 7.0 ± 1.2 cm, *P. leucopus* pairs: 6.9 ± 1.5 cm; *P. maniculatus* individuals: 15.0 ± 1.0 cm, *P. maniculatus* pairs: 16.4 ± 0.9 cm). Thus, pairs of mice dug more burrows than individuals in all three species, but the effect of social context on burrow length varied by species: *P. polionotus* dug significantly longer burrows when digging in pairs, whereas its sister species (*P. maniculatus*) and an outgroup species (*P. leucopus*) did not.

Given that we observed social modulation of burrow length only in *P. polionotus*, we further investigated the effect of trial‐type (i.e., female or male individuals; same‐sex or opposite‐sex pairs) on maximum burrow length in this species. Controlling for mouse ID, we found a significant effect of trial‐type (Fig. 1C; LMM, *F* = 28.52, *P* < 0.001). Specifically, males dug longer burrows than females (planned contrasts, *t* = 2.42, *P* = 0.017) and opposite‐sex pairs dug longer burrows than same‐sex pairs (*t* = 3.44, *P* = 0.001). Male burrows were, on average, 54% longer than female burrows (male: 44.6 ± 2.7 cm, female: 29.0 ± 2.2 cm) and burrows dug by opposite‐sex pairs were, on average, 28% longer than those dug by same‐sex pairs (opposite‐sex: 72.8 ± 6.4 cm, same‐sex: 56.8 ± 3.8 cm).

To control for sex differences and individual variation in burrowing output, we tested whether the burrows dug by different pair‐types (i.e., same‐sex or opposite‐sex) were shorter or longer than expected, given the typical burrow length dug by individuals comprising the pair. For each unique *P. polionotus* pair, we calculated the observed:expected burrow length ratio, where the observed value is the mean burrow length dug by a given pair, and the expected value is the sum of mean burrow lengths dug individually by each member of the pair. Controlling for mouse ID and partner ID, we found that opposite‐sex pairs dug longer burrows than expected compared to same‐sex pairs (Fig. 1D; LMM, *F* = 9.12, *P* = 0.005). The observed:expected burrow length ratio was 0.82 ± 0.05 for same‐sex pairs and 1.18 ± 0.14 for opposite‐sex pairs. This ratio was significantly less than 1 for same‐sex pairs, but statistically indistinguishable from 1 for opposite‐sex pairs (*t*‐tests, FF: *t* = 2.53, *P* = 0.012; MM: *t* = 2.97, *P* = 0.006; FM: *t* = 1.26, *P* = 0.234), indicating that per capita output declines with a same‐sex partner, but is unchanged with an opposite‐sex partner. Together, these data suggest that *P. polionotus* mice alter their burrowing output according to social context (i.e., whether digging with a same‐ or opposite‐sex partner).

### EFFECT OF REPRODUCTIVE STATE ON FEMALE BURROW LENGTH

**Figure 2:**
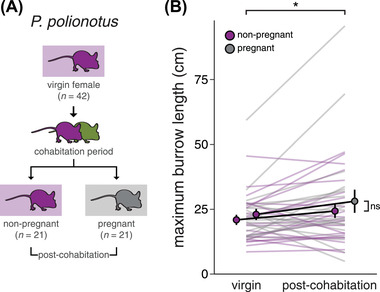
*Peromyscus polionotus* females dig longer burrows after being co‐housed with a male. (A) Experimental design schematic. Individual females were first tested as virgins to determine baseline burrowing output (top). After a period of cohabitation with a conspecific male (middle), females were tested again (bottom). By chance, 21/42 females became pregnant during the cohabitation period. (B) Maximum burrow length dug by females before (left) and after (right) male cohabitation. Each line represents the mean of two trials per individual, per timepoint. *P < 0.05. Error bars represent SEM.

One potential explanation for the observation that opposite‐sex pairs dig longer burrows than same‐sex pairs is that females, when paired with a male, upregulate their digging behavior. Indeed, early studies of burrowing behavior in *P. polionotus* hypothesized that pregnancy may induce increased burrowing output in females (Dawson et al. 1988), as has been documented in other species such as rabbits (Gonzalez‐Mariscal et al. 2007). We therefore tested if *P. polionotus* females that were paired with a male dug longer burrows than those housed only with same‐sex mice. We introduced single virgin females into large, sand‐filled enclosures and, after one overnight period, removed the mouse and cast the resultant burrows. We then co‐housed each female with a conspecific male and re‐tested the female's individual burrowing behavior (Fig. 2A). By chance, half of the females (21/42) became pregnant during the cohabitation period. Notably, we found no effect of virgin burrow length on the probability of becoming pregnant (GLMM, *z* = 0.21, *P* = 0.833). Controlling for mouse ID, we found a significant effect of pairing with a male, but not pregnancy, on the length of the longest burrow dug per trial (Fig. 2B; LMM, cohabitation: *F* = 5.26, *P* = 0.023, pregnancy: *F* = 1.03, *P* = 0.317, cohabitation × pregnancy: *F* = 0.01, *P* = 0.905). *Peromyscus polionotus* females, regardless of pregnancy status, dug longer burrows after cohabitation with a male, with a median increase of 11% over their previous trials (virgin: 22.0 ± 1.4 cm, post‐cohabitation: 26.2 ± 2.6 cm). This contrasts with *P. maniculatus* females, where we found a median burrow length increase of 21% in pregnant females only (Fig. S2, pregnant: *t* = 3.01, *P* = 0.003, non‐pregnant: *t* = 0.93, *P* = 0.355).

We next asked whether this 11% increase in burrowing output by *P. polionotus* females that had been paired with a male could explain the observation that opposite‐sex pairs dig longer burrows than same‐sex pairs (Fig. 1D). To account for this 11% increase, we adjusted the observed:expected burrow length ratio of opposite‐sex pairs by increasing the expected burrow length value for individual females by 11%. However, even with this adjusted expected value, we still found significantly greater observed:expected burrow length ratios for opposite‐sex versus same‐sex pairs (LMM, *F* = 7.01, *P* = 0.012), suggesting that upregulation of digging behavior by paired females alone does not fully explain the longer burrows produced by opposite‐sex pairs.

### NOVEL ASSAY TO MEASURE INDIVIDUAL BEHAVIOR

**Figure 3:**
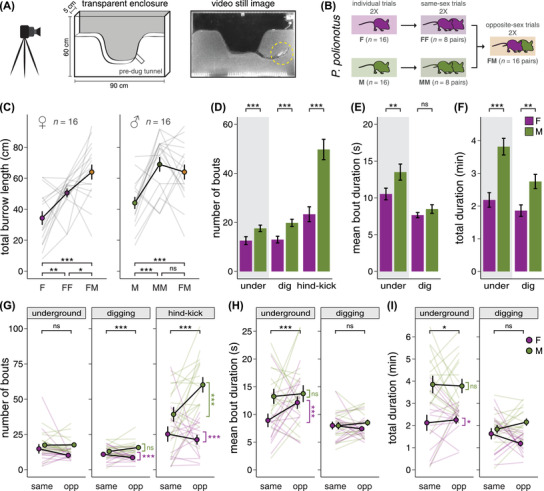
Novel behavioral assay reveals sex and pair‐type differences in burrowing behavior in *Peromyscus polionotus*. (A) Schematic of the narrow, transparent enclosures (left) and still image of two mice digging in tandem (right). (B) Experimental design. Baseline individual burrowing output was first quantified in virgin females and males (left). Mice were then tested, in random order, with a same‐sex (middle) and opposite‐sex (right) partner. (C) Total burrow length produced in three social contexts: individual, same‐sex, and opposite‐sex. Each line represents the mean of two trials per mouse, per context. (D) Number of underground, digging, and hind‐kicking bouts. (E) Mean duration of underground and digging bouts. (F) Total time spent underground and total time spent digging. (D–F) Data reflect the mean of eight observations per individual, pooled across same‐sex and opposite‐sex trials. (G) Number of underground, digging, and hind‐kicking bouts, across same‐sex and opposite‐sex trials. (H) Mean duration of underground and digging bouts, across trial‐types. (I) Total time spent underground and total time spent digging, across trial‐types. (G–I) Data reflect the mean of four observations per individual, per trial‐type. *P < 0.05, **P < 0.01, ***P < 0.001. Error bars represent SEM.

Although our large enclosures are semi‐naturalistic arenas in which mice dig species‐typical burrows, this behavioral assay does not allow us to directly observe digging or determine the relative contributions of individual mice during pair trials. We therefore designed a narrow, transparent sand‐filled enclosure to directly observe their nocturnal, underground behavior (Fig. 3A). In brief, we introduced either one or two mice at the start of the dark cycle into narrow enclosures and recorded 8 hours of overnight video under infrared light and, for all pair trials, manually scored two 10‐minute observation periods per trial (see *Methods* in the Supporting Information). For each observation period, we recorded all burrow entries and exits and specific digging behaviors for both mice (see *Video analysis*). We also measured burrow length using still images from the video taken at the beginning and end of each observation period as well as at the end of the 8‐hour trial (see *Photo analysis*).

First, to first confirm that burrowing behavior in the narrow, transparent enclosures is similar to that observed in the large enclosures, we compared the length of the longest burrow dug per trial by individuals versus pairs of mice. As in the large enclosures, *P. polionotus* pairs dug more burrows (Fig. S3A; LMM controlling for mouse ID and sex, *F* = 23.00, *P* < 0.001) and longer burrows than *P. polionotus* individuals (Fig. S3B; LMM, *F* = 23.29, *P* < 0.001). We also found a significant effect of trial‐type on maximum burrow length (Fig. S3C; LMM controlling for mouse ID, *F* = 9.69, *P* < 0.001). Specifically, males dug longer burrows than females (planned contrasts, *t* = 2.27, *P* = 0.024) and opposite‐sex pairs dug longer burrows than same‐sex pairs (*t* = 2.45, *P* = 0.016). Thus, the effect of social context on burrow length in *P. polionotus* was consistent between our two behavior arenas.

### SEX DIFFERENCES IN BURROW LENGTH

In the transparent enclosures, we used a repeated‐measures design to compare the burrowing behavior of 16 females and 16 males across same‐sex and opposite‐sex trials (Fig. 3B). We first tested all animals as individuals to determine baseline burrowing output and then tested individuals with a same‐sex and opposite‐sex partner, in random order. We found that total burrow length differed significantly between sexes and among trial‐types (LMM controlling for mouse ID, sex: *F* = 5.19, *P* = 0.030; context: *F* = 27.56, *P* < 0.001; sex × context: *F* = 3.52, *P* = 0.032). Compared to individual trials, total burrow length for females increased by 47% in same‐sex trials and 87% in opposite‐sex trials (Fig. 3C; Tukey contrasts, F vs. FF: *t* = 3.09, *P* = 0.007; F vs. FM: *t* = 5.78, *P* < 0.001; FF vs. FM: *t* = 2.74, *P* = 0.019). By contrast, total burrow length for males increased by 51% in pair trials, regardless of pair‐type (Fig. 3C; Tukey contrasts, M vs. MM: *t* = 3.29, *P* = 0.003; M vs. FM: *t* = 3.30, *P* = 0.003; MM vs. FM: *t* = 0.01, *P* = 0.999). These results suggest that females and males may respond differently to changes in social context, but direct observation of individual‐level behavior is necessary to address this question.

### SEX DIFFERENCES IN BURROWING BEHAVIOR

To better understand how females and males behave when digging in pairs, we quantified individual‐level behavior during pair trials. For each mouse in a pair, we recorded underground behavior, digging behavior, and hind‐kicks over four 10‐minute observation periods per mouse, per social context (Fig. S4). We first pooled data across pair‐types to explore sex differences in behavior, using GLMMs with a Poisson link function to analyze number of bouts and LMMs to analyze mean bout duration and total duration (all models included mouse sex and pair‐type as fixed effects and mouse ID, observer ID, trial number, and observation period as random effects). When controlling for social context, we found that males went underground 40% more often, dug 47% more often, and performed 113% more hind‐kicks than females (Fig. 3D; GLMMs, underground: *z* = 4.01, *P* < 0.001; digging: *z* = 4.07, *P* < 0.001; hind‐kick: *z* = 3.94, *P* < 0.001). We also found that, once underground, males stayed in the burrow 28% longer than females (Fig. 3E; LMM, underground: *t* = 2.86, *P* = 0.008), but the mean duration of a digging bout was not significantly different between sexes (LMM, digging: *t* = 0.25, *P* = 0.804). Overall, we found that males spent 74% more time underground and 42% more time digging than females (Fig. 3F; LMMs, underground: *t* = 4.54, *P* < 0.001; digging: *t* = 2.97, *P* = 0.006). Together, these findings are consistent with the observation that individual males dig longer burrows than individual females.

We next examined the relative contributions of individual mice during pair trials. In same‐sex pairs, the division of labor was statistically indistinguishable from 50/50, with each mouse contributing 50.0 ± 3.7% of the total digging duration for the pair (Fig. S3D; *t*‐test, *t* = 0.00, *P* = 1). By contrast, the division of labor was significantly skewed in opposite‐sex pairs, with males contributing 65.8 ± 3.7% of the total digging duration and females contributing 34.2 ± 3.7% (LMM, *F* = 35.29, *P* < 0.001). Because males spend more time digging than females, we might expect, under a simple additive model, that male‐male pairs would spend the most time digging and produce the longest burrows. However, we found that opposite‐sex pairs spent just as much time digging (Fig. S3E; Tukey contrasts, MM vs. FM: *t* = 0.93, *P* = 0.625) and produced burrows that were just as long as male‐male pairs (Fig. S3C; Tukey contrasts, MM vs. FM: *t* = 0.01, *P* = 1). These findings suggest that digging behavior varies with social context in one or both sexes.

### EFFECT OF SOCIAL CONTEXT ON INDIVIDUAL‐LEVEL BURROWING BEHAVIOR

To determine how female and male *P. polionotus* might modulate their behavior according to social context, we compared how individuals behave across same‐sex and opposite‐sex trials. We used GLMMs with a Poisson link function to analyze “number of bouts” and LMMs to analyze “mean bout duration” and “total duration” per 10‐minute observation period and tested for an effect of sex, social context, and sex by social context interaction (Table S1). First, we found no change in the number of underground bouts between same‐sex and opposite‐sex trials (Fig. 3G; LMM, *z* = 1.61, *P* = 0.107). However, females had 21% fewer digging bouts (planned contrasts, *z* = 3.61, *P* < 0.001) and performed 16% fewer hind‐kicks (*z* = 8.23, *P* < 0.001) in opposite‐sex trials, whereas males performed 53% more hind‐kicks in opposite‐sex trials (*z* = 12.46, *P* < 0.001). Second, for females in opposite‐sex trials, we found a 36% increase in the mean duration of an underground bout (Fig. 3H; planned contrasts, *t* = 3.96, *P* < 0.001), but no change in the mean duration of a digging bout between same‐sex and opposite‐sex trials, for either sex (LMM, *t* = 0.25, *P* = 0.804). Third, females spent slightly (6%) more time underground in opposite‐sex trials (Fig. 3I; planned contrasts, *t* = 2.37, *P* = 0.019). In addition, although females spent slightly less time digging in opposite‐sex trials (same: 1.6 ± 0.3 min, opp: 1.2 ± 0.1) and males spent slightly more (same: 1.8 ± 0.2 min, opp: 2.2 ± 0.2), neither trend was significant (Fig. 3I). Together, these results suggest that, although some behaviors (e.g., number of bouts, mean bout duration) may differ slightly by social context, there was no strong difference in total digging duration by individuals in same‐sex versus opposite‐sex trials. Thus, mechanisms other than increased individual effort (i.e., total digging duration) are important for determining pair burrow length.

### DIFFERENCES IN SOCIAL INTERACTION BETWEEN PAIR‐TYPES

**Figure 4:**
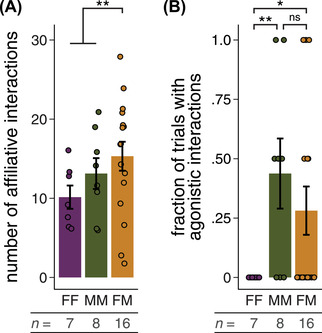
Opposite‐sex *Peromyscus polionotus* pairs show more affiliative and fewer agonistic interactions than same‐sex pairs. (A) Total number of affiliative interactions observed per pair. Each data point represents the sum of four observation periods across two trials. (B) Fraction of trials in which any agonistic behavior was observed. *P < 0.05, **P < 0.01. Error bars represent SEM.

We next explored the frequency of affiliative and agonistic interactions that might indicate differences in social cohesion between same‐ and opposite‐sex pairs. We calculated the cumulative number of affiliative interactions for a given pair across all four observation periods. Opposite‐sex pairs engaged in 31% more affiliative behavior (e.g., allogrooming, huddling) than same‐sex pairs (Fig. 4A; GLM, *z* = 2.69, *P* = 0.007). Next, because agonistic interactions were rarer than affiliative interactions, we calculated the fraction of trials in which any agonistic behavior was observed between mice. We recorded agonistic interactions (e.g., boxing, biting, fleeing) in 44% of male‐male trials, but only 28% of opposite‐sex trials, and no agonistic interactions were observed in any female‐female trial (Fig. 4B; Fisher's exact tests, FF vs. MM: *P* = 0.007; FF vs. FM: *P* = 0.041; MM vs. FM: *P* = 0.339). These results raise the possibility that differences in social cohesion between pair‐types may reflect differences in the propensity to burrow cooperatively.

### DIFFERENCES IN DIGGING EFFICIENCY BETWEEN PAIR‐TYPES

**Figure 5:**
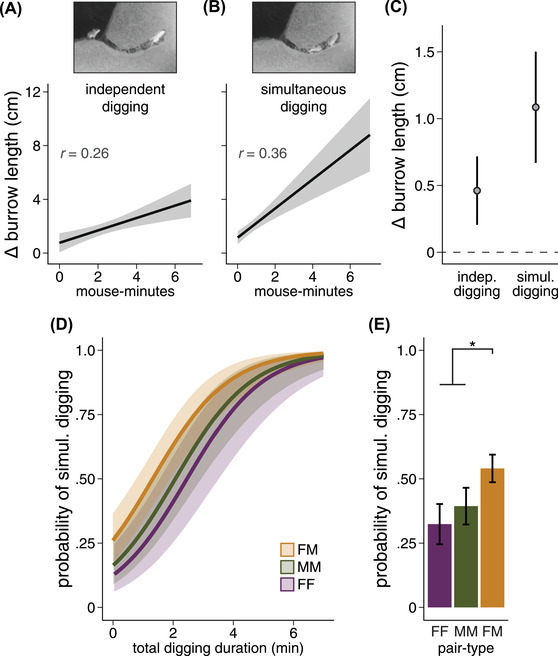
Opposite‐sex pairs are more likely to engage in more efficient simultaneous digging. (A, B) Partial regression plots. (A) Estimated relationship between independent digging duration (i.e., one mouse working at a time) and change in burrow length, controlling for simultaneous digging duration. (B) Estimated relationship between simultaneous digging duration (i.e., both mice working together) and change in burrow length, controlling for independent digging duration. (C) Effect estimates for independent and simultaneous digging duration (in mouse‐minutes) on change in burrow length. (D) Probability of observing simultaneous digging for a range of simulated focal mouse digging durations, given a fixed partner mouse digging duration. (E) Probability of observing simultaneous digging for same‐sex and opposite‐sex pairs. *P < 0.05. Shaded areas and error bars represent 95% confidence intervals.

To understand how opposite‐sex pairs produce longer burrows despite no significant increase in total digging duration, we next tested for differences in digging efficiency between pair‐types. To determine digging efficiency (i.e., burrow extension rate), we compared the change in burrow length over the course of each 10‐minute observation period to the total digging duration observed for both mice during that same period (see *Methods* in the Supporting Information). We distinguished between independent digging (i.e., mice working independently, either temporally or spatially) and simultaneous digging (i.e., mice working together, in the same burrow at the same time) (Video S1). To control for the number of mice, independent and simultaneous digging were both expressed in person‐hours or, more accurately, mouse‐minutes (i.e., time spent digging multiplied by the number of mice digging). To test for an effect of total digging duration on change in burrow length, we used an LMM with independent digging and simultaneous digging as fixed effects and pair ID, observer ID, trial number, and observation period as random effects. As expected, the durations of both independent digging and simultaneous digging were significant predictors of change in burrow length (LMM, independent: *t* = 3.18, *P* = 0.002; simultaneous: *t* = 5.20, *P* < 0.001). Next, to compare the efficiency of these two digging modes, we calculated partial correlation coefficients for both independent and simultaneous digging (Fig. 5A; independent digging: *r* = 0.255, *P* < 0.001, Fig. 5B; simultaneous digging: *r* = 0.356, *P* < 0.001). We found that an additional mouse‐minute of independent digging resulted in an additional 0.46 ± 0.13 cm of burrow length (Fig. 5C). By contrast, an additional mouse‐minute of simultaneous digging resulted in an additional 1.09 ± 0.21 cm of burrow length (Fig. 5C), indicating that simultaneous digging is, on average, 135% more efficient than independent digging, even when controlling for the number of mice digging.

We then asked if the probability of engaging in more efficient simultaneous digging differed between pair‐types. Although both same‐sex and opposite‐sex pairs engaged in both digging modes, opposite‐sex pairs were more likely to engage in simultaneous digging, even when controlling for the total digging duration for both mice (Fig. 5D; GLM, *z* = 2.24, *P* = 0.025). The probability of observing simultaneous digging was significantly higher for opposite‐sex than for same‐sex pairs: 54 ± 5% for opposite‐sex pairs, but only 32 ± 8% for female‐female pairs and 39 ± 7% for male‐male pairs (Fig. 5E). Together, our results suggest that longer opposite‐sex burrows in *P. polionotus* can be explained predominately by an increase in burrowing efficiency—likely mediated by enhanced social cohesion—rather than by an increase in individual burrowing effort.

## Discussion

Here, we show, under controlled laboratory conditions, that social context differentially affects the expression of innate burrowing behavior in three species of *Peromyscus* mice. In two species—*P. leucopus* and *P. maniculatus*—we found no change in burrow length according to social context: the burrows built by individual mice were similar to those built when two mice were assayed together. By contrast, in *P. polionotus*, we found that pairs of mice jointly constructed burrows that were nearly twice as long as those dug by individuals. We further demonstrate, in *P. polionotus*, that opposite‐sex pairs dig longer burrows than same‐sex pairs, driven largely by an increase in digging efficiency (likely due to increased social cohesion) among opposite‐sex pairs. Together, we find evidence of cooperative burrowing behavior in *P. polionotus*, but not its closest relatives.

Collective building behavior has evolved in diverse taxa—from social spiders (Avilés 1997) and eusocial insects (Theraulaz et al. 1998) to communally nesting birds (Cockburn 1998) and cooperatively burrowing mole‐rats (Jarvis et al. 1994). Our observation, that burrows built by *P. polionotus* pairs are nearly twice as long as those built by individuals, is consistent with findings in other species that build collective structures. For example, in most ants and termites, nest volume correlates strongly with colony size (Perna and Theraulaz 2017). This adaptive scaling is mediated by a negative‐feedback process in which insects adjust their digging rate according to traffic flow or the density of workers in the nest (Rasse and Deneubourg 2001). Similarly, bird species in which both sexes contribute to nest building have larger nests than those in which only the female builds the nest (Soler et al. 1998). Thus, in many species, structures built by pairs or groups are larger than structures built by individuals—a trend that we observed in *P. polionotus*, but not *P. leucopus* or *P. maniculatus*. Perhaps even more interestingly, in *P. polionotus*, burrow length differed according to not only the number of diggers (i.e., individuals vs. pairs) but also the composition of the digging pair (i.e., same‐sex vs. opposite‐sex).

Within *P. polionotus*, both same‐ and opposite‐sex pairs cooperatively constructed burrows, but those dug by opposite‐sex pairs were considerably longer. By designing a novel behavioral assay, we could explicitly quantify individual burrowing behavior across different social contexts to determine whether the observed difference in burrow length between same‐sex and opposite‐sex pairs was due to changes in digging duration, digging efficiency, or both. Surprisingly, neither males nor females spent significantly more time digging with an opposite‐sex partner, but instead, longer opposite‐sex burrows arose largely due to increased digging efficiency (i.e., an increased rate of burrow extension). Specifically, opposite‐sex pairs were more likely to dig simultaneously in the same burrow, perhaps in part because they were more socially cohesive and tolerant of a digging partner. Simultaneous digging appeared to be a more efficient mode of burrow extension than two mice digging independently. The simultaneous digging behavior we observed in *P. polionotus* pairs is reminiscent of “chain digging” observed in communally burrowing mammals, such as eusocial mole‐rats (Lovegrove 1989) and group‐living degus (Ebensperger and Bozinovic 2000), and suggests that even distantly related rodent species may have converged upon a common strategy for efficiently excavating shared living space.

One important unanswered question is whether the longer burrows dug by opposite‐sex pairs confer fitness benefits to the pair and/or their offspring. For example, a longer burrow may provide additional protection from predators or buffer against temperature and humidity fluctuations, which are particularly harmful to pups (Berry and Bronson 1992; Bedford et al. 2021). Thus, reproductive pairs may engage in more simultaneous digging to efficiently construct longer burrows, which may, in turn, increase the odds of survival for the pair and/or their pups. Alternatively, longer opposite‐sex burrows could arise simply as a by‐product of increased social cohesion between opposite‐sex partners, with little or no fitness benefits. Notably, the prospect of future reproduction is not strictly necessary for cooperative burrowing behavior in this species: even same‐sex *P. polionotus* pairs constructed longer burrows than individuals, even if not as long as opposite‐sex burrows. Indeed, in the wild, two adult females or two adult males are occasionally found sharing a single burrow (Rand and Host 1942; Blair 1951; Smith 1966), and survival benefits may also accrue to same‐sex pairs that cooperatively construct long burrows. Thus, additional research quantifying the consequences of burrow length variation for both survival and reproduction in the wild is needed to fully untangle the selective pressures (if any) driving the evolution of cooperative burrowing in *Peromyscus*.

Our study revealed that only the monogamous *P. polionotus* demonstrated cooperative burrowing behavior. Although an association between monogamy and cooperation has been previously noted (Dillard and Westneat 2016; Hahn et al. 2021), in this study, these species also differ ecologically, and therefore we cannot rule out the contributions of such factors to interspecific variation in cooperative burrowing. For example, both *P. leucopus* and *P. maniculatus* occupy structured habitats, such as forests and grasslands, and nest in a variety of locations, including rock crevices, brush piles, fallen logs, and tree cavities (Nicholson 1941; Madison et al. 1984; Sharpe and Millar 1990). This diversity of alternative refuges suggests that these mice may only infrequently excavate a burrow *de novo*. In addition, radiotelemetry (Mineau and Madison 1977; Wolff and Hurlbutt 1982; Madison et al. 1984) and nest‐box studies (Nicholson 1941; Wolff and Durr 1986) indicate a general pattern of solitary nesting. Thus, *P. leucopus* and *P. maniculatus* may be facultative, solitary burrowers with few opportunities for cooperative burrowing in the wild. By contrast, *P. polionotus* occupies open habitats, such as fallow fields and sand dunes, and nests almost exclusively in burrows of its own construction (Blair 1951; Weber et al. 2013; Hu and Hoekstra 2017). Moreover, *P. polionotus* often nests in opposite‐sex pairs (Rand and Host 1942; Blair 1951; Smith 1966) and is likely an obligate burrower with ample opportunity for cooperative burrowing in the wild. Thus, in addition to social organization, ecological factors such as habitat type and the availability of alternative nest sites could play a role in the evolution of cooperative burrowing in *Peromyscus*. Further studies examining ecological factors known to modulate behavioral flexibility (e.g., habitat complexity [Leal and Powell 2012], predation risk [Ghalambor et al. 2013], etc.) are necessary to test this hypothesis.

Finally, our results suggest that different *Peromyscus* species may follow different decision rules that govern their burrowing behavior. *Peromyscus maniculatus* and *P. leucopus*, for example, may employ an extrinsic, goal‐oriented strategy in which mice dig until sufficient habitable space has been excavated, resulting in similarly sized burrows regardless of social context (i.e., digging alone or in pairs). By contrast, *P. polionotus* may employ an intrinsic, effort‐oriented strategy in which mice dig until a certain amount of individual effort has been expended, resulting in significantly longer burrows when mice are paired. Our results offer new hypotheses for how different decision rules for building behavior might arise among closely related species.

Overall, our study demonstrates that social context can affect the expression of innate behavior in different ways, even among closely related species. We also show that variation in burrow length can arise in surprising ways: longer opposite‐sex burrows in *P. polionotus* arose due to increased digging efficiency rather than increased individual effort. Together, these findings underscore the importance of combining direct observations of building behavior with measurements of the resulting structures to unravel mechanisms driving behavioral flexibility.

## AUTHOR CONTRIBUTIONS

NLB, JNW, WT, FB, and HEH designed the experiments. NLB, JNW, WT, FB, AK, and RAG performed the experiments. NLB analyzed the data. NLB and HEH wrote the manuscript with input from all authors.

## CONFLICT OF INTERESTS

The authors declare no conflict of interest.

Associate Editor: Dr. C. Moreau

## Supporting information


Supplementary Figures

Supplementary Methods 2

Table S1

**Video S1**: Examples of independent and simultaneous digging in pairs of *P. polionotus* mice
