# Supplementary material for: Interspecific variation in cooperative burrowing behavior by Peromyscus mice
Source: Evol Lett. 2022 Jul 22;6(4):330–40. doi: 10.1002/evl3.293 (PMC9346082; doi:10.1002/evl3.293)
Supplement: Supplementary file 1 — Supplementary Figures [file EVL3-6-330-s004.pdf]

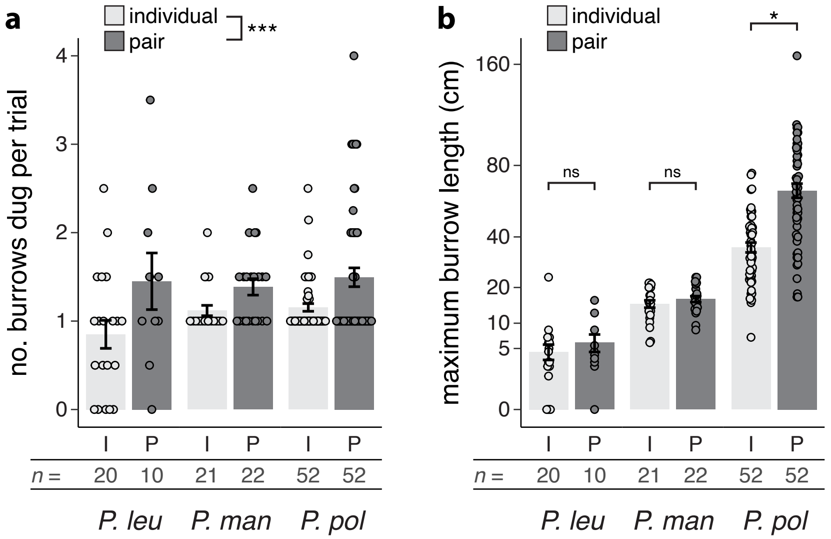

Figure S1 | Comparison of individual and pair burrowing behavior in large, sand-filled enclosures. a) Number of burrows dug in individual (I) and pair (P) trials in three *Peromyscus* species. **b)** Maximum burrow length dug per trial by individuals and pairs of mice. Data are plotted on a square-root scale. Data points represent the mean of 1-7 (average 1.6) trials per individual or unique pair. *$P < 0.05$, ***$P < 0.001$. Error bars represent s.e.m.

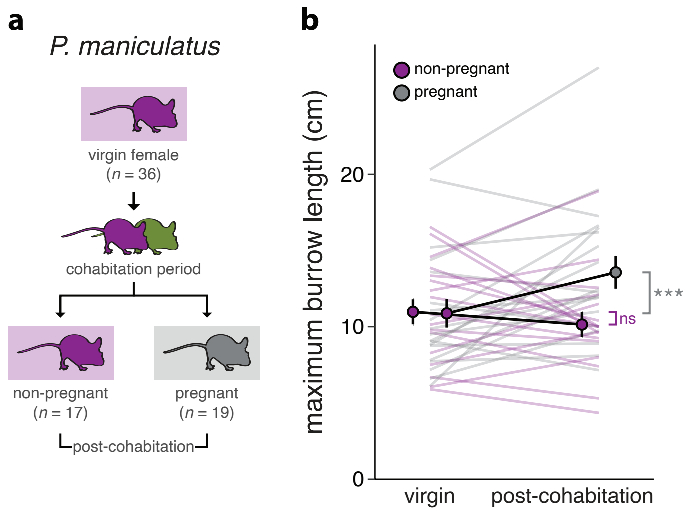

Figure S2 | *P. maniculatus* females dig longer burrows when pregnant. a, Experimental design schematic. Individual females were first tested as virgins to determine baseline burrowing output (top). After a period of cohabitation with a conspecific male (middle), females were tested again (bottom). By chance, 19/36 females became pregnant during the cohabitation period. **b,** Maximum burrow length dug by females before (left) and after (right) male cohabitation. We found a significant interaction between cohabitation and pregnancy on the length of the longest burrow dug per trial (LMM, cohabitation: $F = 1.98$, $P = 0.162$, pregnancy: $F = 1.71$, $P = 0.200$, cohabitation*pregnancy: $F = 7.57$, $P = 0.007$). Using planned contrasts, we found an increase in burrowing output for pregnant females only (pregnant: $t = 3.01$, $P = 0.003$, non-pregnant: $t = 0.93$, $P = 0.355$). 15/19 (79%) pregnant females dug longer burrows after cohabitation with a male, with a median increase of 21% over their previous trials (virgin: 10.9 ± 0.9 cm, post-cohabitation: 13.6 ± 1.1 cm). By contrast, only 6/17 (35%) non-pregnant females dug longer burrows after cohabitation with a male. Each line represents the mean of 2 trials per individual, per timepoint. ***$P < 0.001$. Error bars represent s.e.m.

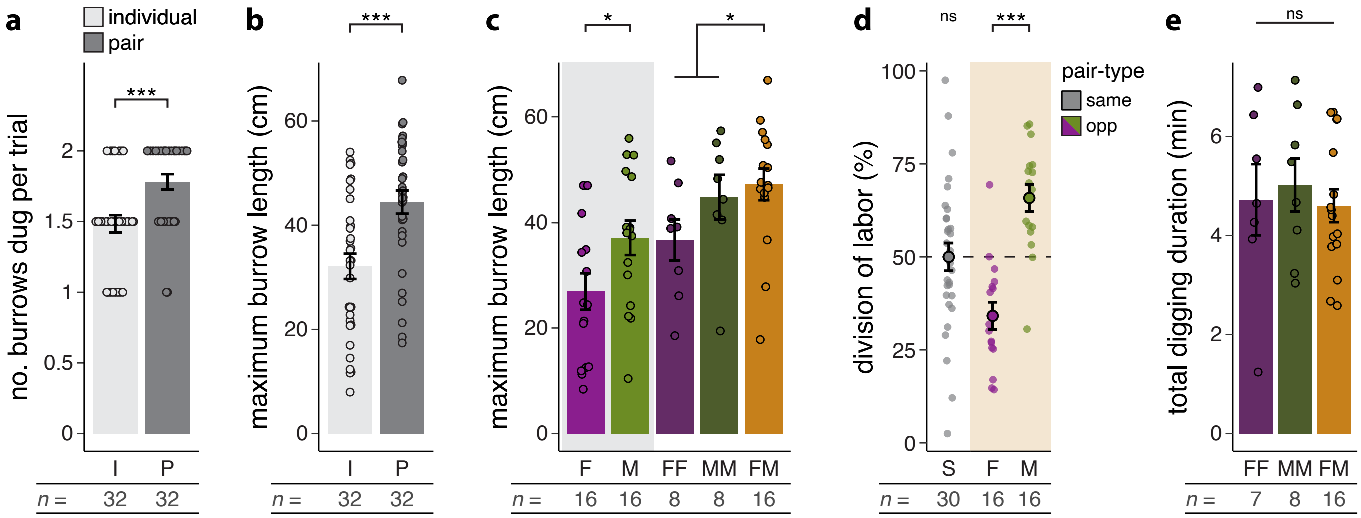

Figure S3 | Summary of *P. polionotus* burrowing behavior in narrow, transparent enclosures. a) Number of burrows dug in individual (I) and pair (P) trials. **b)** Maximum burrow length dug per trial by individuals and pairs of mice. **c)** Maximum burrow length dug per trial, broken down by trial-type. Data points in **a-c** represent the mean of 2 trials per individual or unique pair. **d)** Division of labor in same-sex (S, same) and opposite-sex (opp) pairs. **e)** Total digging duration for same-sex (FF, MM) and opposite-sex (FM) pairs, per ten-minute observation period. Data points in **d-e** represent the mean of 4 observations per pair, over 2 trials. *$P < 0.05$, ***$P < 0.001$. Error bars represent s.e.m.

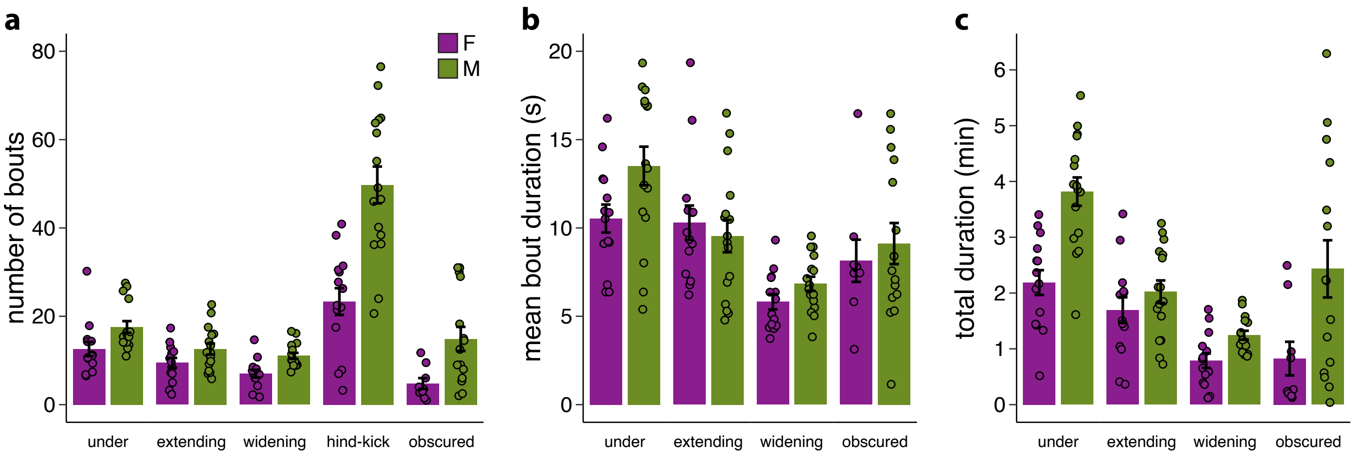

Figure S4 | Sex differences in individual-level burrowing behavior in *P. polionotus*. a-c, Underground and digging behavior, pooled across same-sex and opposite-sex trials, for individual female (n = 16) and male (n = 16) mice. Behavior labels are as follows: "under" (i.e., mouse is underground, in the burrow), "extending" (i.e., mouse is digging at the leading edge of the burrow), "widening" (i.e., mouse is expanding the interior of the burrow), "hind-kick" (i.e., a powerful, expulsive kick that expels loose sand from the burrow), "obscured" (i.e., mouse is underground, but not visible). All behaviors (except hind-kicks) were scored as state events. Hind-kicking bouts were scored as point events. **a,** Number of bouts per observation period. **b,** Mean bout duration, calculated per observation period. **c,** Total duration of behavior per observation period. Data points represent the mean of 8 ten-minute observation periods per individual. Error bars represent s.e.m.
