# Supplementary material for: Interspecific variation in cooperative burrowing behavior by Peromyscus mice
Source: Evol Lett. 2022 Jul 22;6(4):330–40. doi: 10.1002/evl3.293 (PMC9346082; doi:10.1002/evl3.293)
Supplement: Supplementary file 2 — Supplementary Methods 2 [file EVL3-6-330-s003.pdf]

Supplementary Methods

Animal husbandry

We performed experiments with three Peromyscus species: *P. polionotus subgriseus* (PO), *P. maniculatus bairdii* (BW), and *P. leucopus* (LL) obtained from the *Peromyscus* Genetic Stock Center at the University of South Carolina but maintained as outbred colonies at Harvard University. We housed animals in ventilated cages measuring 7.75 x 12 x 6.5" (Allentown Inc., Allentown, NJ), which were furnished with 1/4″ Bed-o'Cobs bedding (The Andersons, Maumee, OH), a 2" square cotton nestlet (Ancare, Bellmore, NY), and a red polycarbonate hut (Bio-Serv, Flemington, NJ). Mice were provided food and water *ad libitum*. Upon weaning at 23 days of age, mice were co-housed in same-sex groups of up to five conspecifics and fed irradiated Prolab Isopro RMH 3000 (LabDiet, St. Louis, MO). We fed all paired adults irradiated breeder chow: PicoLab Mouse Diet 20 (LabDiet, St. Louis, MO). We maintained mice on a 16h light:8h dark cycle at 22°C. The Institutional Animal Care and Use Committee at Harvard University approved all protocols.

Behavioral assays

We measured innate burrowing behavior in two complementary assays under controlled laboratory conditions, allowing us to minimize the effects of other environmental variables (which could also contribute to, for example, interspecific variation in behavioral flexibility).

<u>Large enclosures.</u> We measured burrow architecture as described previously (Weber & Hoekstra 2009; Weber *et al.* 2013; Metz *et al.* 2017). Briefly, at the start of the dark cycle, we introduced individuals or pairs of mice into large PVC boxes (1.2 x 1.5 x 1.1 m) filled with approximately 700 kg hydrated, hard-packed premium play sand (Quickrete, Atlanta, GA). In the enclosures, mice were provided food and water *ad libitum* along with a cotton nestlet. We removed mice from the enclosures after one (pregnancy trials) or two (standard trials) overnight periods. Then, we made casts of the resultant burrows using polyurethane filling foam (Hilti, Schaan, Liechtenstein). Next, a researcher blind to trial identity hand-measured the burrow casts. For each trial, the number of burrows and total length of each burrow was recorded. We also categorized burrow shape as either "simple" or "complex" as described previously (Weber & Hoekstra 2009): *P. leucopus* and *P. maniculatus* burrows are considered "simple" with a short entrance tunnel and terminal nest chamber, whereas *P. polionotus* burrows are considered "complex" with a long entrance tunnel, nest chamber, and escape tunnel. After each trial, we removed all food, feces, nesting material, and disturbed substrate from the enclosure, and then rinsed the enclosure walls with water and turned over the sand to minimize residual odors.

<u>Narrow, transparent enclosures.</u> We also assayed burrowing behavior of mice directly using an acrylic chamber (5 x 90 x 60 cm) with a transparent Plexiglass face (see Fig. 3A). Using a pre-cut mold, we sculpted hydrated sand into two symmetrical 45° hills and excavated an 8 cm tunnel from one randomly selected hill to encourage burrowing in a consistent location. We outfitted the apparatus with an infrared illuminator frame that enabled video recording in the dark. We then introduced individuals or pairs of mice into an enclosure at the start of the dark cycle and removed animals the next morning, recording 8 hours of continuous video during the dark cycle. Mice were provided food and water *ad libitum*. Photographs of the resultant burrows were taken at the end of the trial. Details of photo and video analyses

are provided below. Following each trial, we removed all food, feces, and disturbed substrate and rinsed the enclosure to minimize residual odors.

Experimental design

Effect of social context on burrow length. In the large enclosures, we first measured the burrows of mice from three *Peromyscus* species in individual and pair trials. Sample sizes are indicated in Figure 1. To control for past experience, we randomized the order in which mice were tested in individual, same-sex, or opposite-sex trials. Based on mouse availability, we tested 25 mice once, 43 twice, and 25 more than twice. For pair trials, we tested 34 pairs once, 48 twice, and 2 more than twice. On average, each individual, or each unique pair, was tested 1.6 times (range: 1–7 trials). For all trials, mice were released into the enclosures at the start of the dark cycle and retrieved after two overnight periods (~32 hours).

Effect of reproductive state on female burrow length. To test whether pairing with a male or pregnancy alters burrowing performance, we compared the burrows dug by females before and after being co-housed with a male. First, we tested the burrowing output of virgin females (n = 42 *P. polionotus*, n = 36 *P. maniculatus*) in the large enclosures. Each female was assayed twice with 2 days rest between trials. We then transferred each female to a new cage with a conspecific male. Approximately half of all paired females subsequently became pregnant (21/42 *P. polionotus*, 19/36 *P. maniculatus*). Co-housed females were then tested again twice, as individuals, with 2 days rest between trials. Females were returned to their home cage after each trial and monitored daily for parturition for up to 23 days. To minimize stress to pregnant females, all mice in this experiment were tested for only one overnight period (~8 hours) in the large enclosures.

Effect of social context on individual-level burrowing behavior. To test whether *P. polionotus* mice modulate their behavior according to social context, we used a repeated-measures design to test mice in individual, same-sex, and opposite-sex trials in the narrow, transparent enclosures. To distinguish individuals during pair trials, we shaved a patch of hair from both flanks of one randomly selected member of the pair. Shaving was completed at least a week before the first pairing and was visible over the course of the experiment. We excluded behavioral data from one same-sex pair (FF) in which the markings of the two females were indistinguishable and mouse ID could not be confidently assigned. To quantify baseline burrowing output, we first assayed each individual twice (n = 16 virgin females, n = 16 virgin males). Next, we transferred each individual to a new cage with an unrelated, unfamiliar partner of the same or opposite sex. Pairs were given 2 nights to acclimate before being tested together in the transparent enclosures. We recorded 8 hours of video for each overnight trial, and pairs were then returned to their home cage for 3 nights rest before their second trial. Mice were then re-partnered and the process was repeated. To control for previous experience, we randomly assigned the order in which mice burrowed with a same- or opposite-sex partner. Females were monitored daily for parturition for up to 23 days after the end of the experiment; no females were pregnant at any point during the experiment.

Behavioral analyses

Photo analysis. To measure the length of burrows dug by individuals and pairs of mice in the narrow, transparent enclosures, we took photos of the enclosures at the end of the 8h trial. Each image was measured separately by two researchers, both blind to trial identity, using Fiji image processing software (Schindelin *et al.* 2012). Burrow length measurements were highly correlated between researchers

(Pearson correlation, $r = 0.93$, $P < 0.001$); therefore, we used the mean of these two measurements for all statistical analyses. We also took video stills at the beginning and end of each 10-minute observation period (see *Video analysis*). From these stills, we measured burrow length as described above and subtracted the initial burrow length from the final burrow length to calculate change in burrow length over the 10-minute observation period.

Video analysis. In 75% of trials, the mice had fully completed at least one burrow 3 hours after the start of the dark cycle. Thus, to target the active burrow construction phase, we selected 2 10-minute observation periods, spaced one hour apart: 0:30–0:40 and 1:30–1:40 hours. In 5/62 videos, we did not observe any digging during either of the two observation periods. For these videos, we shifted our two observation periods, still spaced one hour apart, to slightly later in the first 3-hour time window. We accounted for this shift in our statistical analyses by modeling observation period as a random effect (see *Statistics*).

We quantified the behavior of each mouse in a pair using The Observer XT Version 12.0 (Noldus, Leesburg, VA). Each 10-minute observation period (n = 124) was randomly assigned to a researcher blind to trial identity for behavioral scoring according to the following scheme: for each individual, we scored all burrow entries or exits (to calculate time spent underground), as well as extending (i.e., forelimb digging at the growing end of the burrow), widening (i.e., forelimb digging at any other location in the burrow), and hind-kicks (i.e., vigorous, coordinated hindlimb movements that expel loosened sand from the burrow). Sand would occasionally stick to the Plexiglass face of the enclosure, temporarily obscuring the mouse in the burrow; we noted all instances where the mouse was underground, but obscured, thereby precluding our ability to score other behaviors, such as digging or social behavior. We found no effect of social context on how often mouse behavior was obscured (LMM, $t = 1.57$, $P = 0.124$). Time spent underground, time spent obscured, burrow extending, and burrow widening were all scored as state events, whereas hind-kicks were scored as point events. Point events were analyzed as counts only, whereas state events were analyzed as both counts and durations. All events were recorded in Observer to the nearest hundredth of a second. For each pair, we also scored affiliative and agonistic behaviors, which could occur either inside or outside the burrow. We defined affiliative behaviors as beneficial social interactions (i.e., allogrooming, huddling) and agonistic behaviors as aggressive interactions (i.e., boxing, parrying, biting) or submissive behaviors (i.e., freezing, fleeing). Affiliative and agonistic behaviors were scored as state events. Last, to determine how often two mice dug in the same burrow at the same time, we aligned the digging ethograms of the two mice and calculated all instances of overlap. Thus, for each observation period, we calculated the total time spent digging alone and the total time spent digging together.

Altogether, each mouse ($n = 32$) received 80 minutes of direct observation across the entire study: 10 minutes per observation period, 2 observation periods per trial, 2 trials per social context, and 2 social contexts (i.e., same-sex and opposite-sex trials).

Statistics

All statistical tests were performed in R Version 4.1.1 (R Core Team, 2021). We used the *lmer* and *glmer* functions (lme4 package) to run linear mixed-effects models (LMMs) and generalized linear mixed-effects models (GLMMs), respectively. We used the *anova* and *summary* functions (lmerTest package) to determine degrees of freedom, test statistics, and p-values for fixed effect terms. We used the *emmeans*

and *pairs* functions (emmeans package) to determine degrees of freedom, test statistics, and p-values for planned contrasts.

<u>*Effect of social context on burrow length.*</u> Using LMMs, we tested for an effect of species, social context (i.e., individual vs. pair trial), and species by social context interaction on "number of burrows" and "maximum burrow length" dug per trial. To improve normality, we performed a square-root transform on maximum burrow length. We included mouse sex as a fixed effect and mouse ID as a random effect. We calculated the pair: individual burrow length ratio by dividing the length of the average burrow dug by pairs of mice by the length of the average burrow dug by individual mice, excluding 5 *P. leucopus* individuals and 1 *P. leucopus* pair that dug no burrows. Using a Taylor series expansion, we then approximated the variance of each ratio. In *P. polionotus*, we used an LMM to test for an effect of trial-type on maximum burrow length. We modeled mouse ID as a random effect. We then used planned contrasts to test for differences in maximum burrow length between individual females and males, and between same-sex and opposite-sex pairs. Next, we calculated the observed: expected burrow length ratio for each unique pair of mice by dividing the average burrow length dug by a pair of mice by the sum of the average burrow lengths dug by individuals comprising that pair. Using an LMM, we then tested for an effect of pair-type (i.e., same-sex vs. opposite-sex pairs), modeling mouse ID and partner ID as random effects. We tested whether the observed: expected burrow length ratios for FF, MM, and FM pairs were significantly different from 1 using one sample *t*-tests with $\mu = 1.0$.

<u>*Effect of reproductive state on female burrow length.*</u> To first test for an effect of virgin burrow length on the probability of becoming pregnant, we used a GLMM with a binomial link function, modeling mouse ID as a random effect. We then used an LMM to test for an effect of cohabitation with a male, pregnancy, and cohabitation by pregnancy interaction on maximum burrow length, modeling mouse ID as a random effect in both *P. polionotus* and *P. maniculatus*. To improve normality, we log-transformed maximum burrow length. In *P. maniculatus*, we then used planned contrasts to test for an effect of cohabitation with a male on maximum burrow length in mice that became pregnant versus those that did not.

<u>*Sex differences in burrow length.*</u> Using LMMs, we tested for an effect social context (i.e., individual vs. pair trial) on "number of burrows" and "maximum burrow length" dug per trial in the narrow, transparent enclosures (see *Photo analysis*). We included mouse sex as a fixed effect and mouse ID as a random effect. We also used an LMM with mouse ID as a random effect to test for an effect of trial-type on maximum burrow length. We followed up with planned contrasts to test for differences between individual females and males, and between same-sex and opposite-sex pairs. Next, we tested for an effect of trial-type (i.e., individual, same-sex, or opposite-sex), sex, and trial-type by sex interaction on total burrow length. We used an LMM with mouse ID as a random effect and followed up with planned contrasts to test whether females and males respond differently to changes in social context.

<u>*Sex differences in burrowing behavior.*</u> Because we found no effect of sex or pair-type on the ratio of burrow extending to burrow widening (LMM, sex: $F = 0.25$, $P = 0.617$; pair-type: $F = 0.40$, $P = 0.528$), we collapsed these two categories into one general "digging" designation. We then tested for sex differences in underground and digging behavior. We pooled data for individual females and males across same-sex and opposite-sex trials and controlled for social context by including pair-type as a fixed effect in all models. We used GLMMs with Poisson link functions to analyze the number of behavior bouts and LMMs to analyze mean bout duration and total duration. To improve normality, we log-transformed mean bout duration and total duration. All models included mouse sex and pair-type as fixed effects and mouse

ID, observer ID, trial number, and observation period as random effects. To calculate the division of labor in each observation period, we divided the total digging duration for each mouse by the total digging duration for the pair. For same-sex pairs, we tested whether the division of labor was significantly different from 0.5 using a one sample *t*-test with $\mu = 0.5$. We tested whether the division of labor was skewed in opposite-sex pairs using an LMM with sex as a fixed effect and mouse ID, observer ID, trial number, and observation period as random effects. Last, we tested for pair-type differences in total digging duration using an LMM with pair-type as a fixed effect and pair ID, observer ID, trial number, and observation period as random effects. We followed up with Tukey contrasts to test for differences among FF, MM, and FM pairs.

Effect of social context on individual-level burrowing behavior. To assess whether individual mice behave differently in same-sex and opposite-sex trials, we tested for an effect of sex, social context, and sex by social context interaction on underground and digging behavior. We used GLMMs with Poisson link functions to analyze number of bouts and LMMs to analyze mean bout duration and total duration. To improve normality, we log-transformed mean bout duration and total duration. All models included mouse ID, observer ID, trial number, and observation period as random effects (Table S1). We removed all non-significant interaction effects from our models and followed up with planned contrasts to explore significant interaction effects.

Differences in social interaction between pair-types. To quantify differences in social cohesion between pair-types, we calculated the cumulative number of affiliative interactions for a given pair of mice across all 4 observation periods. Using a GLM with a Poisson link function, we tested for an effect of pair-type. Agonistic interactions were more rare than affiliative interactions. We therefore calculated the fraction trials in which any agonistic behavior was observed for a given pair of mice. We used Fisher's exact tests to test for differences among same-sex female-female (FF) and male-male (MM) pairs as well as opposite-sex female-male (FM) pairs.

Differences in digging efficiency between pair-types. To determine digging efficiency, we first measured the change in burrow length over each 10-minute observation period using still images from the video taken at the beginning and end of each observation period (see *Photo analysis*). We then calculated the total duration of both independent digging (i.e., two mice working independently, either temporally or spatially) and simultaneous digging (i.e., two mice working together, in the same burrow at the same time) during the observation period (see *Video analysis*). We expressed the total duration of each digging mode in "mouse-minutes" (i.e., time spent digging multiplied by the number of mice digging).

We tested for an effect of total digging duration on change in burrow length using an LMM with independent digging and simultaneous digging as fixed effects and pair ID, observer ID, trial number, and observation period as random effects. We used partial correlation tests (*pcor.test* function, ppcor package) to determine the correlation between independent digging and change in burrow length, while controlling for simultaneous digging, as well as the correlation between simultaneous digging and change in burrow length, while controlling for independent digging. Because the probability of overlap may increase with digging duration, we calculated the predicted probability of observing simultaneous digging at each value of total digging duration for the focal mouse (which ranged from 0 to 6.83 min), while holding the total digging duration for the partner mouse constant at its mean (1.49 min). We then tested whether the probability of observing simultaneous digging at a given burrow during a given observation period

differed between pair-types. We used a GLM with a binomial link function, including pair-type and total digging duration for both mice as fixed effects.
