# Supplementary material for: Interspecific variation in cooperative burrowing behavior by Peromyscus mice
Source: Evol Lett. 2022 Jul 22;6(4):330–40. doi: 10.1002/evl3.293 (PMC9346082; doi:10.1002/evl3.293)
Supplement: Supplementary file 3 — Table S1 [file EVL3-6-330-s002.pdf]

Table S1A
Model summaries for the number of bouts of underground, digging, and hind-kicking behavior.

Response variable (model)	Fixed effects	Estimate	SE	*z*	*P*
Underground (GLMM)	Intercept	12.13	1.24	11.81	**< 0.001**
	Sex	18.46	1.37	4.01	**< 0.001**
	Context	11.46	1.28	-1.61	0.107
Digging (GLMM)	Intercept	11.82	1.39	7.55	**< 0.001**
	Sex	16.40	1.60	2.31	**0.021**
	Context	9.24	1.48	-3.61	**< 0.001**
	Sex*Context	16.04	1.50	3.83	**< 0.001**
Hind-kicking (GLMM)	Intercept	19.21	1.63	6.07	**< 0.001**
	Sex	24.93	2.01	1.23	0.220
	Context	12.53	1.71	-8.23	**< 0.001**
	Sex*Context	41.16	1.73	13.04	**< 0.001**

GLMMs were run with Poisson link functions. The reference sex is "female" and the reference social context is "same-sex". All models included mouse ID, observer ID, trial number, and observation period as random effects. Statistically significant results are shown in bold. Corresponds to Figure 3G.

Table S1B
Model summaries for the mean bout duration of underground and digging behavior.

Response variable (model)	Fixed effects	Estimate	SE	*df*	*t*	*P*
Underground (LMM)	Intercept	5.79	1.18	11.89	10.54	**< 0.001**
	Sex	11.64	1.39	64.10	4.23	**< 0.001**
	Context	9.93	1.35	215.88	3.99	**< 0.001**
	Sex*Context	3.25	1.42	210.00	-3.11	**0.002**
Digging (LMM)	Intercept	6.71	1.23	2.50	9.04	**0.006**
	Sex	7.11	1.35	32.53	0.66	0.516
	Context	6.83	1.32	193.37	0.25	0.804

LMMs were performed on log-transformed response variables. Back-transformed estimates and standard errors (SE) are reported. The reference sex is "female" and the reference social context is "same-sex". All models included mouse ID, observer ID, trial number, and observation period as random effects. Statistically significant results are shown in bold. Corresponds to Figure 3H.

Table S1C
Model summaries for the total duration of underground and digging behavior.

Response variable (model)	Fixed effects	Estimate	SE	*df*	*t*	*P*
Underground (LMM)	Intercept	49.83	1.31	10.12	14.33	**< 0.001**
	Sex	173.52	1.70	69.75	4.86	**< 0.001**
	Context	85.52	1.65	209.88	2.39	**0.018**
	Sex*Context	26.72	1.79	208.09	-2.01	**0.046**
Digging (LMM)	Intercept	65.87	1.29	6.81	16.28	**< 0.001**
	Sex	124.90	1.60	29.16	2.97	**0.006**
	Context	61.76	1.51	186.68	-0.41	0.683

LMMs were performed on log-transformed response variables. Back-transformed estimates and standard errors (SE) are reported. The reference sex is "female" and the reference social context is "same-sex". All models included mouse ID, observer ID, trial number, and observation period as random effects. Statistically significant results are shown in bold. Corresponds to Figure 3I.
